# Supplementary material for: Centromeres of Cucumis melo L. comprise Cmcent and two novel repeats, CmSat162 and CmSat189
Source: PLoS One. 2020 Jan 16;15(1):e0227578. doi: 10.1371/journal.pone.0227578 (PMC6964814; doi:10.1371/journal.pone.0227578)
Supplement: S1 Fig — (DOCX) [file pone.0227578.s001.docx]

**S1 Fig. The consensus sequences of *CmSat162* and its sequence alignment analyzed by Tandem Repeat Finder**

Indices: 1141832--1144176 Score: 1749

Period size: 162 Copy number: 14.5 Consensus size: 162

1141822 ACCTAGACTA

* * * * *

1141832 TTGCACTTGGGCACTTTGTTGCCATTTCGGGTCATTAAGTCCTTTTATTATCACTTTTTAGTAGG

1 TTGCACTTGGACACTTGGTTGCCATTTCGGGTCGTTAAGTCCTTTT-TTCTCACTTTTTGGTAGG

* ** * * **

1141897 TTATTG-GCTAAGAAAGCTCACACTTAAGCA--CAATTTGGTTGTCTTT-ACAACTTAGAAACCT

65 TTA-TGAGTTGGGAAAGCTCACACTTAAGCATCCTA-TT-GTTGT-TTTGACAGCTTAGGCACCT

** ** ** ** *

1141958 CGT-AGATTCTACTTAGGTTCATTTAACACTATAGTTG

126 TTTGA-ACACTACTTAGGTTGGTTTGGCACTATGGTTG

* * *

1141995 TTGCACTTGG-CAC-TGAGTTGCCATCTCGGATCGTTATGTCCTTTTTTCTCACTTTTTTGGTAG

1 TTGCACTTGGACACTTG-GTTGCCATTTCGGGTCGTTAAGTCCTTTTTTCTCAC-TTTTTGGTAG

* * * * * *

1142058 GTTTTGAGTTGGAAAATCTCATACTTAAGCATCCTATTGTTGTTTTGACATCTTAGGTACCTTTT

64 GTTATGAGTTGGGAAAGCTCACACTTAAGCATCCTATTGTTGTTTTGACAGCTTAGGCACCTTTT

* * * **

1142123 AAACACTACTTAGATTGGTTTAGCACTACAGTTG

129 GAACACTACTTAGGTTGGTTTGGCACTATGGTTG

* ** * * *

1142157 TTGCACTTGTGTA-A-TTGATTTTCATTTCAGGTCGTGAAGTTCTTTTTTCTCA-TTATTTGGTA

1 TTGCACTTG-G-ACACTTGGTTGCCATTTCGGGTCGTTAAGTCCTTTTTTCTCACTT-TTTGGTA

** ** * *

1142219 GGTTATGAGTTGGGAAAGCTCATGCTTAAGCATTGT-TTGGTTGTCTTT-ATAGCTTAGG-ATAC

63 GGTTATGAGTTGGGAAAGCTCACACTTAAGCATCCTATT-GTTGT-TTTGACAGCTTAGGCA-CC

* *

1142281 TTTTGGACACTACTTAGGTTGGTTTGACACTA-GGATTG

125 TTTTGAACACTACTTAGGTTGGTTTGGCACTATGG-TTG

* * * ** *** * *

1142319 TCGTACTTGAACACTTGGTTGTTATTTTTTGTTGTTAAGTCCTTTTTTTCTCACTTTTTAGTAGG

1 TTGCACTTGGACACTTGGTTGCCATTTCGGGTCGTTAAGTCC-TTTTTTCTCACTTTTTGGTAGG

* * * * * *

1142384 TTATGAGCTGGGAAAGCTCACACTTAAGCATCATATTGTTGTCTTGACA-CTACAGACACCTTTG

65 TTATGAGTTGGGAAAGCTCACACTTAAGCATCCTATTGTTGTTTTGACAGCT-TAGGCACCTTTT

* * * * *

1142448 GAACACTACTTAGGTTAGTTTGGTACAACGGTTA

129 GAACACTACTTAGGTTGGTTTGGCACTATGGTTG

*** ** * **

1142482 TTGCACTTGGACACTTGGTCATCATTTCGGGTTATTAAGTCCTTTTTTCTCACTCTTTGGTAATT

1 TTGCACTTGGACACTTGGTTGCCATTTCGGGTCGTTAAGTCCTTTTTTCTCACTTTTTGGTAGGT

* * * **

1142547 TATGAGTTGGGAAAGCTCACACTTAAGAATCCTATTGTTATCTCT-ACAGCTTAGGCACCTCATG

66 TATGAGTTGGGAAAGCTCACACTTAAGCATCCTATTGTTGT-TTTGACAGCTTAGGCACCTTTTG

* * * * **

1142611 AACACTACTTAGGTTTGTTTAGAATTATATTTG

130 AACACTACTTAGGTTGGTTTGGCACTATGGTTG

* * *

1142644 TTGCAGTTGGACACTTGGTTGCCATTTCGGGTCGTGAAGT-CTTTGTTTCTCACTCTTTGGTAGG

1 TTGCACTTGGACACTTGGTTGCCATTTCGGGTCGTTAAGTCCTTT-TTTCTCACTTTTTGGTAGG

* * * ** * * * * *

1142708 TTATGAGTTAGGAAAGCTCACACATATGCATTGT-TTGGTTCTTTTTACAACTTAGGCATCTTAT

65 TTATGAGTTGGGAAAGCTCACACTTAAGCATCCTATT-GTTGTTTTGACAGCTTAGGCACCTTTT

* * * *

1142772 GGACACTACTTACGTTGGTTTGGCATTATAGTTG

129 GAACACTACTTAGGTTGGTTTGGCACTATGGTTG

* * * *

1142806 TTGTACTTGGGCACTTGGTTGTCATTTCGGGTCGTTAAGT-CTTTTATTCTCACTTTTTTGTAGG

1 TTGCACTTGGACACTTGGTTGCCATTTCGGGTCGTTAAGTCCTTTT-TTCTCACTTTTTGGTAGG

* * * * * *

1142870 TTATAAGTTGGGAAAGTTCACACTTAAGCAT-CTCATTGTTATCTTGACACCTTAGGCACCCTTT

65 TTATGAGTTGGGAAAGCTCACACTTAAGCATCCT-ATTGTTGTTTTGACAGCTTAGGCACCTTTT

* *

1142934 GAACACTACTTAGGTTTGTTTGGCACTAGGGTTG

129 GAACACTACTTAGGTTGGTTTGGCACTATGGTTG

* * * * * *

1142968 TTGTACTT-GAGCACTTGATTGTCATTTCGGGTCATAAAGTCTTTTTTTTTCTCACTTTTTGGTA

1 TTGCACTTGGA-CACTTGGTTGCCATTTCGGGTCGTTAAGTC--CTTTTTTCTCACTTTTTGGTA

* ** * * ** * *

1143032 GGTTATGAGTTGGGAAAGCTTACACTTAAGCATTGTTTTGTTGTCTCCATATCTTAGGCACC-TT

63 GGTTATGAGTTGGGAAAGCTCACACTTAAGCATCCTATTGTTGTTTTGACAGCTTAGGCACCTTT

* * *

1143096 TGGACACTACTTAGGTTGGTTTGGCACGAGGGTTG

128 TGAACACTACTTAGGTTGGTTTGGCACTATGGTTG

* * * * ** *

1143131 TTGCACTAGGACACTTGGTTTCCATATCGGGTCATTAAGTCCTTTTTTCTCACCATTTCGTAGGT

1 TTGCACTTGGACACTTGGTTGCCATTTCGGGTCGTTAAGTCCTTTTTTCTCACTTTTTGGTAGGT

* * * * * * * *

1143196 TATAAGTTGTGAAAGCTCACATTTAAGCATCCTATTGTTATTTTGGCACCTTAGACACCTTTGGA

66 TATGAGTTGGGAAAGCTCACACTTAAGCATCCTATTGTTGTTTTGACAGCTTAGGCACCTTTTGA

* * *

1143261 ACACTACTTAAGTAT-GTTTGGCACTAGGGTTA

131 ACACTACTTAGGT-TGGTTTGGCACTATGGTTG

** *

1143293 TTGCACTTGGACACTTGGTTGCCATTTTAGGTCGTTAAGTCCTTTTTTCTCGCTTTTTGGTAGGT

1 TTGCACTTGGACACTTGGTTGCCATTTCGGGTCGTTAAGTCCTTTTTTCTCACTTTTTGGTAGGT

* * * * *

1143358 TATAAGTCGGGAAAGCTCACACTTAAGTATCCTATTGTTGTCTTT--CAGCTTAGGTA-GTTCAT

66 TATGAGTTGGGAAAGCTCACACTTAAGCATCCTATTGTTGT-TTTGACAGCTTAGGCACCTT--T

** ** * *

1143420 T-AACACTACTTAGGTTTTTTTAACACTATAGTTA

128 TGAACACTACTTAGGTTGGTTTGGCACTATGGTTG

* ** * *** * * *

1143454 TTGTACTTGGGTACTTGGTTATCC-TTTCCAATCATGAAGTCCTTTTTGCTCA-TATTTTGGTAG

1 TTGCACTTGGACACTTGGTT-GCCATTTCGGGTCGTTAAGTCCTTTTTTCTCACT-TTTTGGTAG

* * ** * * *

1143517 GTTATGAGTTGGGAAAGCTCACATTTAACCATTGT-TTGGTTATCTTT-ACAGCTTAGGCATCTC

64 GTTATGAGTTGGGAAAGCTCACACTTAAGCATCCTATT-GTTGT-TTTGACAGCTTAGGCACCTT

* * * ** *

1143580 ATGGACAATACTTAGGTTTATTTGACACTATGGTTG

127 TTGAACACTACTTAGGTTGGTTTGGCACTATGGTTG

* * * *

1143616 TTGCACTTGGACACTTGGTTGCTATTTCGGATCATTAAGTCCTTTTTTCTCACTTTGTGGTAGGT

1 TTGCACTTGGACACTTGGTTGCCATTTCGGGTCGTTAAGTCCTTTTTTCTCACTTTTTGGTAGGT

* * * * * *

1143681 TATGAGTT-GGAAAGCTAACACTTAAGTATCCTATTATTGTCTTGACACCTTAAGCACCTTTTGA

66 TATGAGTTGGGAAAGCTCACACTTAAGCATCCTATTGTTGTTTTGACAGCTTAGGCACCTTTTGA

* * * *

1143745 ATACTACTTTGGTTTGTTTTGGCACTAAGGTTG

131 ACACTACTTAGG-TTGGTTTGGCACTATGGTTG

* * * * * * * *

1143778 TTACGCTT-GAGCACTTGGTCGCCATTACGAGTCGTTAATTCATTTTTTCTCACTTTTTGGTAAG

1 TTGCACTTGGA-CACTTGGTTGCCATTTCGGGTCGTTAAGTCCTTTTTTCTCACTTTTTGGTAGG

* ** ** * * * *

1143842 -T-TGAGTTGGGAAAGCTCAAACTTAAAAATTGT-TTGATTGTCTTT-ATAGCTTAGACATCTTA

65 TTATGAGTTGGGAAAGCTCACACTTAAGCATCCTATTG-TTGT-TTTGACAGCTTAGGCACCTTT

* * ** *

1143903 TGCACATTACTTAGGTT-GTTT-G-AC-ATTATAG

128 TGAACACTACTTAGGTTGGTTTGGCACTATGGTTG

** * * *

1143934 TTGCACTTGGACACTTAATTGCCATTTCAGGTCGTTAGGTCCTTTTTTTTCTCATTTTTTTGGTA

1 TTGCACTTGGACACTTGGTTGCCATTTCGGGTCGTTAAGTCC--TTTTTTCTCA-CTTTTTGGTA

* * ** *

1143999 GGTTATAAGTTGGGAATGCTCACACTTAAGCATTGT-TTGATTGTCTTT-ACAGCTTAGGCATCT

63 GGTTATGAGTTGGGAAAGCTCACACTTAAGCATCCTATTG-TTGT-TTTGACAGCTTAGGCACCT

* * * * * *

1144062 TTTGGACACTATTTAGGTTGGTTTTGCCCTATAGTTA

126 TTTGAACACTACTTAGGTTGGTTTGGCACTATGGTTG

* * * * *

1144099 TTGCACTTGGGCACTTGGTTACTATTTCAGGTCGTTAAGT--TTTTTTCTCACTTTTTTGGTATG

1 TTGCACTTGGACACTTGGTTGCCATTTCGGGTCGTTAAGTCCTTTTTTCTCAC-TTTTTGGTAGG

1144162 TTATGAGTT-GGAAAG

65 TTATGAGTTGGGAAAG

1144177 GTTTTCCACT

Statistics

Matches: 1732, Mismatches: 379, Indels: 145

0.77 0.17 0.06

Matches are distributed among these distances:

156 31 0.02

157 4 0.00

158 11 0.01

159 13 0.01

160 25 0.01

161 396 0.23

162 893 0.52

163 237 0.14

164 80 0.05

165 42 0.02

ACGTcount: A:0.22, C:0.17, G:0.20, T:0.41

Consensus pattern (162 bp):

TTGCACTTGGACACTTGGTTGCCATTTCGGGTCGTTAAGTCCTTTTTTCTCACTTTTTGGTAGGT

TATGAGTTGGGAAAGCTCACACTTAAGCATCCTATTGTTGTTTTGACAGCTTAGGCACCTTTTGA

ACACTACTTAGGTTGGTTTGGCACTATGGTTG
