# Supplementary material for: Centromeres of Cucumis melo L. comprise Cmcent and two novel repeats, CmSat162 and CmSat189
Source: PLoS One. 2020 Jan 16;15(1):e0227578. doi: 10.1371/journal.pone.0227578 (PMC6964814; doi:10.1371/journal.pone.0227578)
Supplement: S2 Fig — (DOCX) [file pone.0227578.s002.docx]

**S2 Fig. The consensus sequence of *CmSat189* and its sequence alignment analyzed by Tandem Repeat Finder**

Indices: 1876224--1876740 Score: 827

Period size: 189 Copy number: 2.7 Consensus size: 189

1876214 ACAATATGTC

1876224 AAGTGTATTAAATCTATCAAGTGTATCAGCACATCATAACAAGTGTATCAACAATATATAAAGTG

1 AAGTGTATTAAATCTATCAAGTGTATCAGCACATCATAACAAGTGTATCAACAATATATAAAGTG

* *

1876289 TATCATGCTTAGTGCATCAAGTATATTTAATTGATTGAGTGTATCAACAGTTGAACAAGTGTGTC

66 TATCATGCTTAGTGCATCAAGTATATTTAATTGATTGAGTGTATCAACAGTTGAGCAAGTGTATC

* * *

1876354 AACAATATACCAAGTGTTTCAAATTAATAATATGTATCAAGAAAGTTTAGCAAGTGATT

131 AACAACATACCAAGTGTTTCAAACTAATAATATGTATCAAGAAAGTTTAACAAGTGATT

* *

1876413 AAGTGTATTAAATCTATCAAGTATATCAGCACATCATAACAAGTGTATCAACAGTATATAAAGTG

1 AAGTGTATTAAATCTATCAAGTGTATCAGCACATCATAACAAGTGTATCAACAATATATAAAGTG

* * * * ** *

1876478 TATCATGCTTCGTGGATGAAGTGTATTTTGTTGATTGAGTGTATCAACAGCTGAGCAAGTGTATC

66 TATCATGCTTAGTGCATCAAGTATATTTAATTGATTGAGTGTATCAACAGTTGAGCAAGTGTATC

*

1876543 AACAACATACCAAGTGTTTCAAACTAATAATATGTATCAAGGAAGTTTAACAAGTGATT

131 AACAACATACCAAGTGTTTCAAACTAATAATATGTATCAAGAAAGTTTAACAAGTGATT

* * * * *

1876602 AAGTGTATTAAATCTATCAAGTGTATCAGAAAACCATAATAAGTGTATCAACAATACATAAAGTG

1 AAGTGTATTAAATCTATCAAGTGTATCAGCACATCATAACAAGTGTATCAACAATATATAAAGTG

* * *

1876667 TATCATGCTTAGTGCATCAAGTATATTTAATTGGTTGAGTGTATCAACAATTGAGCAATTGTATC

66 TATCATGCTTAGTGCATCAAGTATATTTAATTGATTGAGTGTATCAACAGTTGAGCAAGTGTATC

1876732 AACAACATA

131 AACAACATA

1876741 TATTGGTGTA

Statistics

Matches: 296, Mismatches: 32, Indels: 0

0.90 0.10 0.00

Matches are distributed among these distances:

189 296 1.00

ACGTcount: A:0.39, C:0.13, G:0.16, T:0.32

Consensus pattern (189 bp):

AAGTGTATTAAATCTATCAAGTGTATCAGCACATCATAACAAGTGTATCAACAATATATAAAGTG

TATCATGCTTAGTGCATCAAGTATATTTAATTGATTGAGTGTATCAACAGTTGAGCAAGTGTATC

AACAACATACCAAGTGTTTCAAACTAATAATATGTATCAAGAAAGTTTAACAAGTGATT
