# Supplementary material for: Centromeres of Cucumis melo L. comprise Cmcent and two novel repeats, CmSat162 and CmSat189
Source: PLoS One. 2020 Jan 16;15(1):e0227578. doi: 10.1371/journal.pone.0227578 (PMC6964814; doi:10.1371/journal.pone.0227578)
Supplement: S3 Fig — For Cmsat162, sequence from nt1141769 to nt1144288 of LN681816 was used for dot plot analysis whereas sequence from nt1875724 to nt1877241 was used for CmSat189. The consensus sequences of both CmSat162 and CmSat189 are listed in S2 Table. (DOCX) [file pone.0227578.s003.docx]

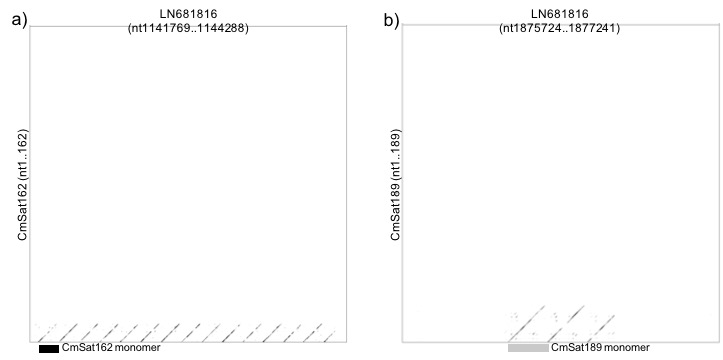


**S3 Fig**. *CmSat162* (A) and *CmSat189* (B) repeat organization in *Cucumis melo* genomic scaffold sequence (accession number: LN681816). For *Cmsat162*, sequence from nt1141769 to nt1144288 of LN681816 was used for dot plot analysis whereas sequence from nt1875724 to nt1877241 was used for *CmSat189*. The consensus sequences of both CmSat162 and CmSat189 are listed in Table S2.
