# Supplementary material for: Centromeres of Cucumis melo L. comprise Cmcent and two novel repeats, CmSat162 and CmSat189
Source: PLoS One. 2020 Jan 16;15(1):e0227578. doi: 10.1371/journal.pone.0227578 (PMC6964814; doi:10.1371/journal.pone.0227578)
Supplement: S1 Table — (PDF) [file pone.0227578.s004.pdf]

S1 Table. **SatDNA repeats on melon DNA sequence "LN681816" analyzed by Tandem Repeat Finder ver. 4.09**

Indices	Period Size	Copy Number	Consensus Size	Percent Matches
1138926-1139180	115	2.2	114	80
1141832-1144176*	162	14.5	162	76
1141832-1144202	325	7.4	325	76
1142032-1144216	487	4.5	482	78
1876224-1876740*	189	2.7	189	90

*SatDNA repeats used in this study
