# Supplementary material for: Centromeres of Cucumis melo L. comprise Cmcent and two novel repeats, CmSat162 and CmSat189
Source: PLoS One. 2020 Jan 16;15(1):e0227578. doi: 10.1371/journal.pone.0227578 (PMC6964814; doi:10.1371/journal.pone.0227578)
Supplement: S2 Table — (DOCX) [file pone.0227578.s005.docx]

**S2 Table**. The consensus sequences of *CmSat162* and *CmSat189* acquired from LN681816 DNA sequence analyzed by Tandem Repeat Finder ver. 4.09

| Gene | Sequence |
| --- | --- |
| *CmSat162** | TTGCACTTGGACACTTGGTTGCCATTTCGGGTCGTTAAGTCCTTTTTTCTCACTTTTTGGTAGGTTATGAGTTGGGAAAGCTCACACTTAAGCATCCTATTGTTGTTTTGACAGCTTAGGCACCTTTTGAACACTACTTAGGTTGGTTTGGCACTATGGTTG |
| *CmSat189** | AAGTGTATTAAATCTATCAAGTGTATCAGCACATCATAACAAGTGTATCAACAATATATAAAGTGTATCATGCTTAGTGCATCAAGTATATTTAATTGATTGAGTGTATCAACAGTTGAGCAAGTGTATCAACAACATACCAAGTGTTTCAAACTAATAATATGTATCAAGAAAGTTTAACAAGTGATT |

*: The monomer lengths of these sequences were determined based on bioinformatic analysis.
