# Supplementary figures and images for: Centromeres of Cucumis melo L. comprise Cmcent and two novel repeats, CmSat162 and CmSat189
Source: PLoS One. 2020 Jan 16;15(1):e0227578. doi: 10.1371/journal.pone.0227578 (PMC6964814; doi:10.1371/journal.pone.0227578)

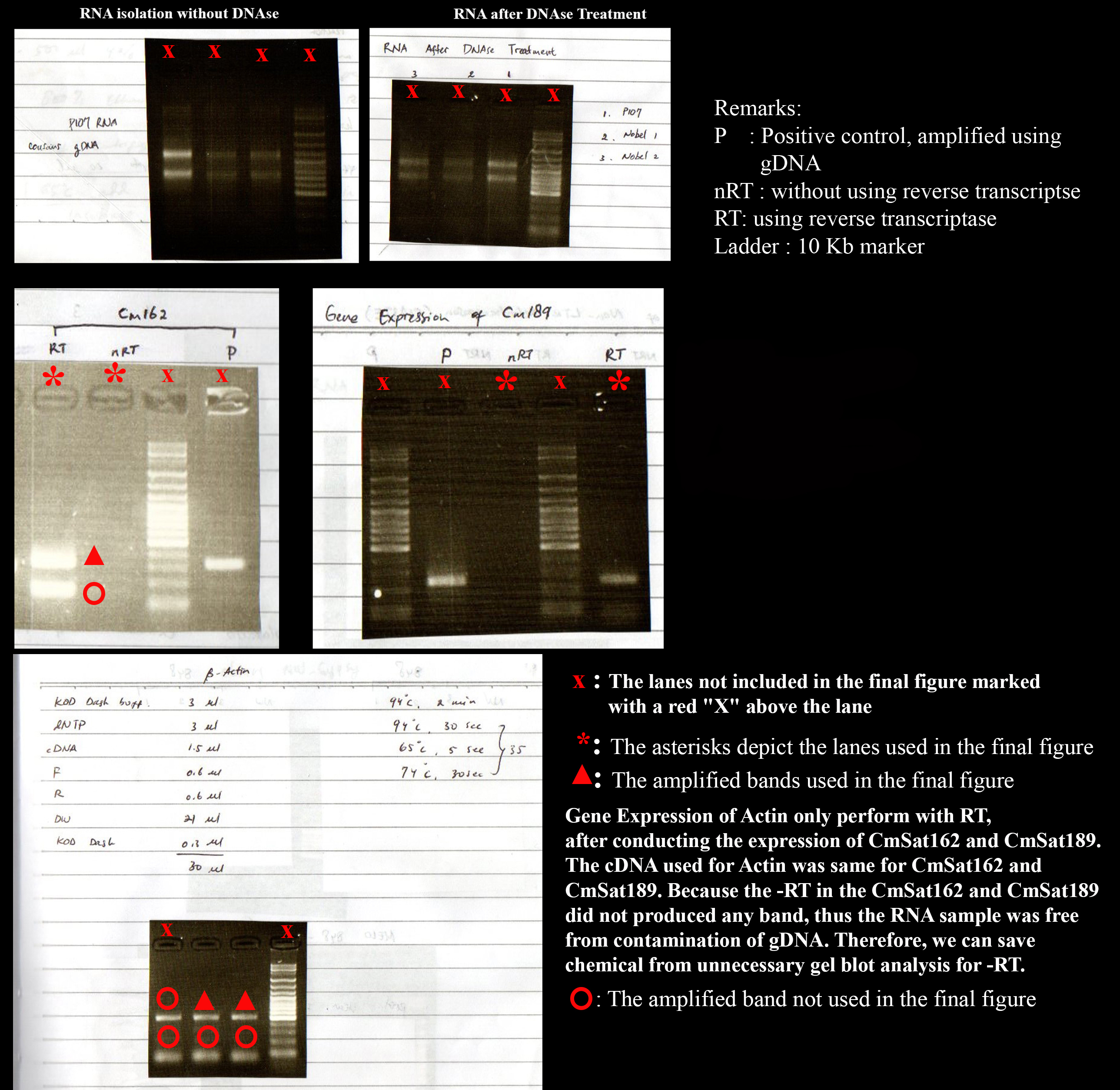

Supplement: S1 Row Images — (JPG) [file pone.0227578.s006.jpg]
