# Supplementary material for: Centromeres of Cucumis melo L. comprise Cmcent and two novel repeats, CmSat162 and CmSat189
Source: PLoS One. 2020 Jan 16;15(1):e0227578. doi: 10.1371/journal.pone.0227578 (PMC6964814; doi:10.1371/journal.pone.0227578)
Supplement: Attachment — Submitted filename: Response to reviewers.docx [file pone.0227578.s007.docx]

**Response to reviewers**

**Reviewer 1**

| **Comments** | **Responses** |
| --- | --- |
| The manuscript “Centromeres of Cucumis melo L. comprises Cmcent and two novel repeats, CmSat162 and CmSat189” by Setiawan and others describes the identification of two new tandem repeat sequences.These two sequences largely colocalize with the established centromeric sequence Cmcent at the primary constriction. Melon centromere evolution is particularly interesting as species of this genus show evolutionary centromere repositioning events. These events are common in animals, but not in plants. What the role of centromeric sequences are in centromere repositioning events are remains unknown. Expanding our knowledge of what types of sequences are associated with centromeres, and whether these sequences are limited to the centromere or also found along the chromosome arms is important for understanding the evolutionary dynamic behavior of these fast evolving sequences. This work provides novel insights in satellite DNA in the very interesting Cucumis genus. |  |
| The authors used scaffolds to identify new tandem repeats sequences using TRF. Could the authors provide the TRF settings used? | The “Basic” mode of TRF setting [https://tandem.bu.edu/trf/trf.submit.options.html] was used to identify new tandem repeats sequences: the alignment parameters (match, mismatch, indels) = (2, 7, 7), minimum alignment score to report repeat = 50, maximum period size = 500 and maximum TR array size (bp, millions) = 2. .This setting is the default parameters set by TRF. |
| Could the authors provide a reasoning why they chose to use scaffolds to identify novel tandem repeats, rather than WGS data? WGS data allows you to assemble from sequences that not only present in the scaffolds. Melters et al 2013 Genome Biology used this very approach to identify hundreds of candidate centromere tandem repeats. This approach would also provide the authors with an estimated quantity of the sequence identified. | Melters et al. (2013) used TRF to isolate tandem repeat DNAs from Pac Bio and Sanger sequences. The authors also used PRICE to analyze Illumina reads to obtain tandem repeat DNAs. Both approaches used by Melters et al. (2013) require computer workstation or cluster with high Random-Access Memory (RAM) and personnel with certain bioinformatic background. We chose to use TRF to analyze published scaffolds rather than WGS data, because it was easy and rapid method to identify tandem repeat in the published scaffolds. |
| Were the CmSat162 and CmSat189 sequences found the genome of other Cucumis species? A BLASTn search would give a quick indication. | Using BLASTn search, only the CmSat162 sequences found in the cucumber genome, but no sequence similarity of CmSat189 was found in other genomes of cucumis. |
| What were the sizes of the FISH probes? In our hands, monomer sized FISH probes allow for very specific hybridization, whereas longer probes might hybridize with less specificity. | The size of the PCR product of CmSat162 is 311 bp, and the size of the PCR product of CmSat189 is 244 bp. We used these size of PCR products to produce probes and we used dimer instead of monomer for FISH. |
| At the end of the introduction, the authors introduce the histone variant CENH3. For clarity, please specify that nucleosomes containing CENH3 instead of H3 mark the functional centromere. | This issue has been corrected in the manuscript. |
| In the final paragraph of the introduction, it is unclear what question the authors set out to answer in this manuscript. | The final paragraph has been modified, and we point out in this manuscript that we identified two new satellite DNA in melon as centromeric probes which previously only limited to Cmcent and there is no other report except Cmcent. |
| Figure 1: did the authors also check for CmSat162 signal on pachytene chromosomes from ‘Baladewa’ and ‘Ivory F1 hybrids’? Ideally, this is reported as well. | No, we did not check for Cmsat162 signal on pachytene chromosomes from ‘Baladewa’ and ‘Ivory F1 hybrids’. We only checked on P90. |
| Figure2: did the authors also check for CmSat189 signal on ‘Baladewa’ and ‘P90’ chromosomes? Especially the karyotype ideogram is very informative. Showing this for all three cultivars and for both novel tandem repeats would provide a great resource for the field. | No, we did not check for CmSat189 signal on ‘Baladewa’ and ‘P90’ prometaphase chromosomes. We only checked on ‘Ivory F1 hybrids’. |
| Do CmSat162 and CmSat189 colocalize on (pachytene) chromosomes of the three tested cultivars? | We only performed FISH of CmSat162 and Cmcent on the pachytene of P90, and it colocalize. We did not perform FISH of CmSat162 and CmSat189 on the three tested cultivars. Since CmSat189 was colocalized with Cmcent on prometaphase of ‘Baladewa’. Therefore, it maybe colocalize with CmSat162. |
| It is very interesting that transcripts of CmSat162 and CmSat189 were found by sqPCR. Were these transcripts cell cycle specific? Can this be determined from leave tissue? In addition, the -RT control, ideally a control with and without RNase, and with and without DNase is done to guarantee that no DNA contamination persisted. | The transcript of CmSat162 and CmSat189 were obtained from leave tissue. The total RNA was isolated from leaves. To guarantee that no DNA contamination in the RNA sample, we use DNase treatment prior to use reverse transcriptase. The RNA that we used for +RT and -RT were treated with DNase and that free from gDNA. We used DNase I from Nippongene [https://www.nippongene.com/siyaku/product/modifying-enzymes/dnase1/dnase-rt-grade.html] |

**Reviewer 2**

| This paper describes two satellites from melon that the authors show are cytologically located at the centromeres, and other locations. They also show that these satellite sequences are  transcribed. |  |
| --- | --- |
| The presentation of the satellite sequences is somewhat confusing. The authors identify the 162 bp and 189 bp satellites using Tandem Repeat finder on a genomic scaffold. According to Table S1, the 162 bp repeat family and the 189 bp repeat family show 76% and 90% identity, respectively. They describe cloning the repeats using primers that amplify dimers of the repeats, but the primers given in the methods only partially match the satellite monomer sequences given in Table S2, making it unclear what sequences were actually cloned, and whether the monomers in Table S2 are particular examples of monomers or are consensus sequences. In Figure 3, comparing dimers of the two satellites to each other and to the previously identified satellite Cmcent, there is limited symmetry between the two halves of the dimers, especially for the 162 bp satellite, suggesting considerable divergence in the sequences of the two monomers used in the display. The authors also fail to mention how long Cmcent is and whether a monomer or dimer is displayed. They do mention in the Introduction that Sat107 is 352 bp long and is centromeric, but they do not identify this with Cmcent. There needs to be a better explanation of how the primers, monomers, and dimers relate to each other. The authors should state in the text in addition to Table S1 the degree of conservation of the two satellite families, and whether the sequences in Table S2 are consensus sequences or not. It might be useful to have a supplementary figure showing the relationship of the primers, monomer sequence in Table S2, and the dimer sequences in Figure 3 to the sequences amplified from the genomic scaffold. | We identify the tandem repeat using TRF, then we make a dimer of the consensus sequence of the repeat. The primer was designed and we did the PCR using those primers. The size of the PCR product of CmSat162 is 311 bp, and the size of the PCR product of CmSat189 is 244 bp. The amplified bands were labeled using PCR labeling and those were hybridized on melon chromosomes, and we clone the amplified bands from PCR using pGEM-T easy vector.  The sequences listed in Table S2 are the consensus monomer sequences of CmSat162 and CmSat189 that identified using TRF. The repeat organizations of both repeats are shown in Figure S3.  The melon centromere repeat clone (Cmcent) was provided by Koo Dal-Hoe (Koo et al., 2010) to the co-author of this manuscript, Dr. Shinji Kikuchi. The monomer of Cmcent is displayed in Figure 3.  pSat107 and Cmcent shared high sequence homology as shown in the figure below and Figure 3d.    The relationship of the primers, monomer sequence in Table S2, and the dimer sequences in Figure 3 to the sequences amplified from the genomic scaffold have been shown in amended Figure 3. |
| Do the authors know whether one or both strands of the satellites are transcribed? Were the primers used for sqPCR the same as those used  for gene amplification? | Both strands of the satellites are transcribed in leaf tissues and the primers were used for semi quantitative PCR (sqPCR), same as those used for gene amplification. The primers used for both sqPCR and gene amplification were mentioned in the method section. |
| It would be helpful at some point for the authors to mention that there are currently no functional data or CENH3 ChIP data to identify the centromere sequences in Cucumis, and the identification is solely by FISH. | This has been added in the end of discussion section. |
| Minor points  The title is ungrammatical (Centromeres…comprises). | This has been corrected |
| Line 3: Not all centromeres are associated with repetitive sequences. Though this is common in plants and animals, it is not true of many single-celled eukaryotes. | This sentence has been eliminated from the abstract to prevent the confusion. |
| Line 16: I think the authors mean “portion”, not “proportion”. | This has been corrected |
| In Figures 2d and 3d, it would be helpful to the reader to number the chromosomes 1-12 at the bottom of the figures. | The numbers has were added under the chromosome arrangements. |
